# Bibliography

**Published:** 1903-04

**Authors:** 


### Bibliography.

Long's Dental Materia Medica and Therapeutics. A Text-Book of Dental Materia Medica and Therapeutics for Students and Practitioners of Dentistry. By Eli H. Long, M.D., Professor of Materia Medica and Therapeutics in the Medical and Dental Departments of the University of Buffalo, N. Y. In one octavo volume of three hundred and twenty-one pages, with twenty-four illustrations, including eighteen full-page colored plates. Lea Brothers & Co., Philadelphia and New York, 1903.

If there is a text-book more needed than any other in dentistry it is a work on Materia Medica properly prepared for the students in dental colleges. When this book was taken up for review it was with the hope that within its pages this desire might be realized; but as these were read, while there was found much to praise there was an equal amount to condemn.

The dedication of the book is an evidence of a lack of tact, as it is a violation of historical evidence. It is "To the memory of William T. G. Morton, M.D., who first made known surgical anæsthesia," etc. This at once rouses a feeling of resentment that Horace Wells should be entirely ignored, but then Wells was not a medical-graduate, and that explains, perhaps, the peculiar dedication.

"The author's conception of the book that is needed to-day is one that will serve three purposes,—viz., *first,* to treat fully all remedies that belong properly to the special field of dental medicine; *second,* to discuss briefly the action and application of the most important general remedies, emphasizing those whose action may avail in dental diseases and emergencies; and *third,* to furnish matter for reference that will cover all ordinary demands of the dental student and practitioner as to general remedies, their preparation, doses, and uses."

Chapter I. is taken up with the consideration of "Drugs and Medicines," and is well arranged. The next, Chapter II., is "Remedies: Their Classification and Definitions." Chapter III., "Administration of Medicines," follows, and then, in Part II., Chapter IV., begins the consideration of drugs under "Depletives, Counterirritants, Escharotics," etc.

This classification is very objectionable when presented to the dental student. It may be necessary in medical teaching, and hence the medical author assumes that it is impossible to arrange a work on Materia Medica in any other form. All the books upon this subject recently published have this serious objection. The consideration of drugs for dental use should be general, without omitting their peculiar properties. When placed under special heads, cross reference is a necessity, and this, in study, becomes a serious annoyance.

One of the radical errors of the book, in the opinion of the reviewer, is the omission of any description of the origin or derivation of the drugs described. The author starts out with a general description, and then follows this with the therapeutic value of the drug under consideration. This is all very well for one familiar with the subject, but the undergraduate will find himself wondering whence this or that drug is derived. The therapeutic descriptions are better than usual, but at times the lack of dental knowledge is painfully apparent. This is in evidence in the description of the effect of arsenic. The author says, "Within a varying time after the application, usually several hours, the patient will experience pain, first gnawing, later throbbing, in character, which will continue until the pulp is destroyed." That this is all wrong requires no argument to the well-instructed dentist. Where pain continues for several hours the arsenic is simply producing increased irritation upon an already irritated pulp, and should be at once removed and sedatives and analgesics applied.

Under Argenti Nitras the author states: "It has long been used to cauterize wounds that are probably infected. . . . It does not penetrate deeply, therefore cannot be relied upon to destroy the infected tissue." This is altogether wrong, for the reviewer several years ago proved by experiment that this generally supposed action of silver nitrate was erroneous. It was proved that this agent was one of the escharotics that *did* penetrate deeply; in fact, the limit of penetration could not be decided.

In writing of soaps the author says of Castile, "Its chief dental use is in dentifrices." The use of soap for this purpose should be discouraged. While an alkali may be beneficial in neutralizing acidity it is a serious question whether in the course of years it has not a deleterious effect upon the organic basis substance of the teeth. This opinion was expressed years ago by a chemist of reputation, and at the time was combated by the reviewer, but experience has tended to confirm the opinion that continued application does affect organic matter, although apparently protected by enamel.

As usual, there is found under Sedatives and Dentition the old and well-worn advice that, "If dentition is found to be abnormal or difficult and teeth are nearly ready to appear, the gums may be scarified, as mentioned before. But cutting the gums over teeth that are not likely to appear for several months is questionable practice, as the tissue will rapidly heal," etc. The reviewer would kindly suggest that the author would do well to spend some time in a well-appointed dental histological laboratory before he publishes a second edition, for a more absurd paragraph could hardly be penned. In the first place, scarification will not avail, and in the second, deep lancing at all stages of development is not only good practice, but absolutely essential to permit the tooth to advance gumward and thus prevent pressure upon the undeveloped root and pulp. It is, however, hopeless to expect some medical men, and an occasional dentist, to rise to the level of intelligence on this subject. The reviewer would recommend the author to read Dewees, on "The Medical Treatment of Children," published by Carey, Lea & Blanchard in 1834, where he will find, among other good things, on page 329, this wise paragraph:

"And though the tooth cut upon may be yet remote from the surface, still the operation may be of the greatest possible advantage by dividing the membrane, now severely put upon the stretch, and from which the whole irritation proceeds. . . . The disturbance of the system is quieted from the moment the gum is divided."

The diagrams in colors showing the action of drugs on various organs are both novel and very satisfactory. They objectively appeal to the reader, and must impress the student's mind better than any method with which the reviewer is familiar.

While there is much to object to in the arrangement of the book, there is much that is of real value in it, and it is regretted that the author failed, apparently, to seek good dental criticism before publishing. If the book reaches a second edition the author should remember that students need detail; their knowledge can never be safely taken for granted. The derivation of drugs, therefore, is as important as the therapeutics, and the obsolete method of considering these under distinct heads, when dealing with dental undergraduates, should be abandoned. This done, it will be acceptable to dental colleges.

The chapter on Prescription Writing is of special value, and the "Index of Drugs" is a valuable contribution to the general practitioner.

The book is prepared in the well-known and much appreciated style of Lea Brothers & Co.

A Pocket Text-Book of Anatomy. By Wm. H. Rockwell, Jr., M.D., Assistant Demonstrator of Anatomy, College of Physicians, Columbia University, New York. In one 12mo volume of six hundred pages, with seventy illustrations. Lea's Series of Pocket Text-Books. Edited by Bern B. Gallaudet, M.D. Lea Brothers & Co., Philadelphia and New York.

The author of this manual of Anatomy has endeavored to present a text-book to "Prevent the confusion often experienced by the student when, in using a manual for review which is not in harmony with the larger work to which he is accustomed, he meets with differences in the text, even if these differences are unimportant from a practical stand-point."

The text-books of anatomy, in the form of manuals, are quite numerous, and, doubtless, they supply a demand notwithstanding that the larger works, especially Gray's, are in many respects more valuable for careful study. This manual follows Gray, and is therefore an abridged form of the latter, and in this respect a convenience to the student.

The only criticism that can be made is that the illustrations are too limited. While the student is not supposed to derive his anatomy from these, they are a material aid in review, and their absence in some portions lessens the value of the book. This is noticeable in Osteology, especially in the description of the skull.

While the illustrations in other parts are more frequent, an increase would add materially to the value of the book. To the earnest student of anatomy this will not be a serious objection, and the advantage of compactness and clearness of description will counterbalance this defect.

PREFACE AND SUMMARY OF THE WORK OF DR. C. E. DE M. SAJOUS, PHILADELPHIA, ON THE DUCTLESS GLANDS.

The first volume prepared on this subject by the author will soon be published. The book, as a whole, will be noticed as soon as it appears. The author has sent advance sheets of the Preface and Summary of Contents to this journal, from which quotations are made.

The views of this author have created a wide-spread interest in medical and lay circles and the final publication of these in full will be received with unusual satisfaction. In order that our readers may have some idea of Dr. Sajous's work, it may be best to permit him to tell the story in two or three paragraphs quoted from the Preface. After describing preliminary work, he says, "The thyroid gland, the anterior pituitary, and the adrenals were thus found to be functionally united; *i.e.*, to form an autonomous system, which we termed the 'adrenal system.'

"Further investigation in this direction showed that the action of thyro-iodine upon the anterior pituitary body represented that of any poison introduced into the blood-stream. In other words, it became evident that, instead of acting directly upon the blood or cellular elements, poisons either stimulated or depressed the functional activity of the adrenal system, thus increasing or reducing the production of adrenal secretion, and, therefore, of oxidizing substance in the plasma. Radical changes in prevailing doctrines as to the manner in which general infections or other forms of poisoning produced their effects on the organism thus seemed to impose themselves. . . . We were thus led to conclude that what are now considered as symptoms of infection or poisoning are all manifestations, more or less severe, of *overactivity or insufficiency of the adrenal system*. Indeed, *the physiological action of remedies was also traced to the anterior pituitary body,* the governing centre of the system."

Further on he says, "The *posterior pituitary body,* far from being the insignificant vestigial organ it is generally thought to be, was found by us . . . to stand second in importance only to its mate, the anterior pituitary body. Indeed, it proved to be the chief functional centre of the nervous system, its numerous groups of neurons forming the starting-point, a highly specialized centre of a single class of nerves."

Again: "Briefly, our inquiry seems to us to have shown that the adrenal system is the source of the secretion which, with the oxygen of the air, forms the oxidizing substance of the blood plasma. It has also revealed, we believe, the origin and mode of distribution of the bodies with which this oxygen directly or indirectly combines; *i.e.*, peptones, myosinogen, fibrinogen, hæmoglobin, and myelin, to insure the continuation of life and the efficiency of all organic functions. Finally, it has suggested that, in addition to these agencies, all leucocytes and, under certain circumstances, the plasma, contain a protective agency, trypsin, which, with Metchnikoff's phagocytic cells, serves to destroy micro-organisms and convert the toxins and other albuminoid poisons into harmless products. Considered jointly, these various factors seem to us to represent the aggregate of vital phenomena."

It will be necessary, before reaching conclusions as to the value of Dr. Sajous's very interesting work, to await the publication before alluded to. The author's well-known and remarkable labors in medical literature entitle this work, when it appears, to special consideration.

